# Intestinal parasite infections in immigrant children in the city of Rome, related risk factors and possible impact on nutritional status

**DOI:** 10.1186/1756-3305-5-265

**Published:** 2012-11-20

**Authors:** Laura Manganelli, Federica Berrilli, David Di Cave, Lucia Ercoli, Gioia Capelli, Domenico Otranto, Annunziata Giangaspero

**Affiliations:** 1 Azienda Ospedaliera Universitaria Policlinico Tor Vergata, Viale Oxford 81, Rome, Italy; 2 Dipartimento di Medicina Sperimentale e Chirurgia, University of Tor Vergata, Via Montpellier 1, Rome, Italy; 3 Istituto Zooprofilattico Sperimentale delle Venezie, Viale dell’Università 10, Padova, Legnaro, Italy; 4 Dipartimento di Sanità pubblica e Zootecnia, University of Bari, Valenzano, Bari, Italy; 5 Dipartimento di Scienze Agrarie, degli Alimenti e dell’Ambiente, University of Foggia, Via Napoli 25, Foggia, Italy

**Keywords:** Intestinal parasites, Risk factors, Nutritional status, Immigrant children, Developed country

## Abstract

**Background:**

Parasitic diseases can represent a social and economic problem among disadvantaged people - even in developed countries. Due to the limited data available concerning Europe, the aims of the present study were to evaluate the presence of parasites in immigrant children and the risk factors favouring the spread of parasites. Subsequently, the possible correlation between nutritional status and parasitic infections was also investigated.

**Findings:**

A convenience sample of two hundred and forty seven immigrant children (aged 0–15) attending the Poliambulatorio della Medicina Solidale in Rome was examined. Data were collected using structured questionnaires, and parasitological and anthropometric tests were applied. Chi-squared test and binary logistic multiple-regression models were used for statistical analysis.

Thirty-seven children (15%) tested positive to parasites of the following species: *Blastocystis hominis, Entamoeba coli, Giardia duodenalis, Enterobius vermicularis, Ascaris lumbricoides* and *Strongyloides stercoralis*. A monospecific infection was detected in 30 (81%) out of 37 parasitized children, while the others (19%) presented a polyparasitism. The major risk factors were housing, i.e. living in shacks, and cohabitation with other families (p<0.01). Children classified in the lower height Z-scores had a significantly greater prevalence of parasites (30.9%) than the others (p<0.01).

**Conclusions:**

This study shows that parasite infection in children is still quite common, even in a developed country and that children’s growth and parasitism may be related. Extensive improvements in the living, social and economic conditions of immigrants are urgently needed in order to overcome these problems.

## Findings

### Background

Parasitic infections in children are an important public health issue, particularly in developing countries where social and economic deprivation, poor hygienic conditions and warm climates favour the spread of intestinal parasites. Worldwide, 3.5 billion people are affected by intestinal parasites, and 450 million people, mostly children, present clinical symptoms [1]. Parasites lead to malabsorption and chronic blood loss in children, with long-term effects on their physical (height-weight) and cognitive development [2-4]. Parasitic diseases represent a social and economic problem in developing countries [5-9]. Malnutrition makes the children more vulnerable to intestinal parasites, which in turn leads to a poor nutritional status, creating a synergistic relation impairing growth. Disadvantaged groups in industrialized countries like immigrants and/or nomads are at risk for parasites [10,11].

Considering that the population of children from 0 to 14 y.o is 8,439,916 in Italy, the percentage of immigrant children now stands at 11% of the total. The official statistics obviously do not include illegal immigrants [12].

In view of the lack of information concerning European countries, the aims of the present study were to evaluate the presence of parasites in immigrant children and the risk factors favouring the spread of parasites. Subsequently, the possible correlation between nutritional status and parasitic infections was also investigated.

## Methods

The study was carried out at the Poliambulatorio della Medicina Solidale e delle Migrazioni in Rome which offers free health assistance to immigrants and/or deprived people, most of them without a residence permit.

From January 2008 to September 2010, a convenience sample of 247 children (aged 0 to 15) was accurately visited and registered their personal data and medical history (e.g., gender, age, weight, height, reported illnesses), number of family members, country of origin, travel, household and environmental conditions.

Every child was examined, and anthropometric analysis and parasitological tests were performed. Briefly, children were weighed without clothes (weight scales SECA 725 with a sensitivity of 50 g and a capacity of 150 kg) and their height was recorded (ruler measuring up to 2,000 mm). The following indicators were used: H/A and W/A Z-scores for children up to 10 years of age, and only H/A Z-scores for children from 11 to 15 years old; values ranging from −2 to + 2 Z-scores were considered, according to the parameters provided by WHO [13,14]*.* Nutritional indicators were calculated using "WHO Anthro” PC Software, version 2, 2007 (*http://www.who.int/entity/childgrowth/software/who_anthro_pc.pdf*).

Faeces were collected for three consecutive days by providing a child’s parent/guardian with three labelled flasks and instructions on procedures in four different languages (English, French, Romanian and Italian).

Additionally, children with specific symptoms (i.e. anal pruritus, abdominal pain, irritability and restlessness) at the clinical examination were subjected to scotch test to detect *Enterobius vermicularis* eggs. Faecal samples were subjected to macro- and microscopical examination for intestinal parasites in triplicate, and examined by direct fresh iodine staining and concentration. Briefly, a Para-Fix kit containing formalin 10% was used for stool preservation; cysts, eggs and larvae were detected by examining faecal samples using the Sed-Connect closed concentration kit (Medical Chemical Corporation) containing formalin, ether and ethilacetate; samples were also stained using the Ziehl-Neelsen modified technique, for *Cryptosporidium* oocyst detection. An immunofluorescence test was used to confirm the diagnosis of *Cryptosporidium* and *Giardia* (Kit MERIFLUOR® *Cryptosporidium/Giardia*, Meridian Diagnostic, Cincinnati, OH, USA).

The parent/s or legal guardians of children participating in the study signed a written consent form. All research protocols followed the principles of the Helsinki Declaration and its subsequent modification, as well as those of Italian National Law no. 675.1996 concerning the protection of personal data.

The prevalence differences in relation to nutritional status were analysed using the Chi-squared test. In order to evaluate possible risk factors associated with parasite prevalence, the epidemiological data were analysed using binary logistic multiple-regression models [15]. The parasitological status of each child (positive/negative) was used as a dependent variable. The following independent variables were applied to the model: age (coded as 1=<5 years; 2=6-10; 3=11-15), gender, country of origin (European and others), time spent in Italy (> or < than 1 year), travel (yes/no), housing in apartments or shacks (i.e. brick houses with running water and toilet facilities or else roughly built houses in camps with water and toilets serving the whole population), cohabitation of the child with other family groups (yes/no). Statistical analyses were performed using SPSS for Windows version 13.0.

## Results

The study population (i.e., gender, age, provenance, time spent in Italy, travel history, housing, cohabitation, nutritional status) is reported in Table 1. Of these 247 children, 181 (73.2%) were of European origin, including 163 (65.9%) from Romania, 45 (18.2%) were from Africa, 10 (4.0%) from Asia, and 11 (4.4%) from South America. All children were vaccinated and none of the investigated children had any chronic diseases, severe pathological conditions or notable childhood infectious diseases, which could affect immunological status. Most children did not present any clinical signs, but 21 (8.5%) had diarrhoea, and 20 (8%) abdominal pain.

**Table 1:** Prevalence (P) for parasites in relation to epidemiological data and significant differences

**Epidemiological data**		**Examined**	**Positive**	**P**	**Significance**
*Gender*	females	118	15	12.7%	χ^2^ = 0.912
	males	129	22	17.1%	P = 0.219
*Age (years)*	< 5	158	16	10.1%	χ^2^ =24.57
	6-10	67	10	14.9%	P < 0.01
	11-15	22	11	50.0%	
*Height (Z-score)*	z < −1	55	17	30.9%	χ^2^ = 16.76
	−1<z<1	138	18	13.0%	P < 0.01
	z > 1	54	2	3.70%	
*Weight (Z-score)*	z < −1	40	8	20.0%	χ^2^ = 5.550
	−1<z<1	132	16	12.1%	P = 0.062
	z > 1	50	2	4.0%	
*Provenance*	European	181	27	14.9%	χ^2^ =0.002
	other	66	10	15.2%	P = 0.553
*Time in Italy*	< 1 year	85	22	25.9%	χ^2^ = 12.096
	> 1 year	162	15	9.3%	P < 0.01
*Travel*	no	178	27	15.2%	χ^2^ = 0.018
	yes	69	10	14.5%	P = 0.534
*Housing*	shack	21	10	47.6%	χ^2^ = 19.198
	apartment	226	27	11.9%	P < 0.01
*Living with other families*	no	170	18	10.6%	χ^2^ = 8.258
	yes	77	19	24.7%	P < 0.01

The nutritional status of these children was good in 138 cases (55.8%), while 54 (21.8%) of the children were overweight or obese, and 55 (22.2%) presented growth rates below normal standard values, although none of the children showed H/A and W/A Z-scores < −2 or > +2.

Thirty-seven children (15%) resulted positive for protozoans (i.e., *Blastocystis hominis, Entamoeba coli* and *Giardia duodenalis*) or helminths (i.e., *Enterobius vermicularis, Ascaris lumbricoides* and *Strongyloides stercoralis*) with a monospecific (81%) or multiple infections (19%) (Table 2).

**Table 2:** Prevalence of parasite infection in 247 children aged 0–15 in Italy

	**No.s of positive children**	**Prevalence (%)**	**Prevalence among positives (%)**
**Monoparasitism**	30	12.15	81.1
**Polyparasitism**	7	2.83	18.9
**Total positive**	37	15.00	100
**Parasite species**			
*Blastocystis hominis*	19	7.69	51.35
*Entamoeba coli*	12	4.86	32.43
*Giardia duodenalis*	9	3.64	24.32
*Enterobius vermicularis*	3	1.21	8.11
*Ascaris lumbricoides*	1	0.40	2.70
*B. hominis* + *G. duodenalis*	1	0.40	2.70
*B. hominis* + *E. coli*	2	0.81	5.41
*E. coli* + *G. duodenalis*	3	1.21	8.11
*B. hominis + S. stercoralis*	1	0.40	2.70

Of 37 children who tested positive for parasites, 32 (86.5%) did not present clinical symptoms related to their condition, but 3 (8.1%) suffered from abdominal pain (one with *B. hominis* and two with *E. vermicularis*), and two (5.4%) presented diarrhoea (one with *G. duodenalis + E. coli*, and one with *B. hominis*). None of the positive children showed clinical signs of anaemia. Evalutation of children’s nutritional status revealed that none of the parasitized children suffered from acute malnutrition (W/A), whereas 2 (5.4%) were overweight and 17 (46%) were significantly affected by chronic malnutrition (H/A) (p<0.01). Of the children with chronic malnutrition, 11 had a monospecific infection (i.e., *B. hominis* n= 7, *G. duodenalis* n = 2, *A. lumbricoides* n = 1, *E. coli* n = 1), and six presented mixed infections (*B. hominis* and *E. coli* n = 2; *G. duodenalis* + *E. coli* n = 2*; G. duodenalis + B. hominis*, *B. hominis* + *S. stercoralis* n = 1, respectively). Two of the overweight children had *G. duodenalis*. Regular medical treatment was provided for the children found to be infected.

Risk factors associated with the parasite prevalence showed that children living in shacks are 2.7 times more likely to be parasitized than others living in apartments (p<0.01), whereas increasing age and cohabitation with other people were less relevant (Table 3). Interestingly, the risk of being positive for parasites decreased according to the time spent in Italy, probably due to the reduced exposure to parasites compared with the country of origin. This effect was particularly evident in those children living in apartments (data not shown), who showed a significant reduction of positivity after a longer stay in Italy (6.1% vs 23.1%, respectively, p<0.01), compared to children living in shacks where parasite positivity is similar regardless to the time spent in Italy (57.1% vs 42.9%).

**Table 3:** Significant risk factors associated with parasite prevalence

**Risk factors**^**a**^	**P value**	**Odds ratio**	**95% C.I. lower-upper**	
Increasing age^b^	0.011	2.060	1.180	3.596
Time in Italy	0.002	0.488	0.308	0.775
Housing	0.001	2.776	1.522	5.062
Cohabitation	0.009	1.739	1.146	2.640

^a^ variables were categorized as shown in Table 1.

^b^ the OR represents the predicted change in odds for a unit increase of the age category (shown in Table 1).

The evaluation of prevalence differences in relation to nutrition revealed that children classified in the lower height Z-scores presented a significantly higher prevalence of parasites (30.9%) than the others (p<0.001) (Table 1).

### Discussion and conclusions

Among the immigrant communities examined, a relevant percentage of children (15%) were infected by parasites, compared to the lower parasite prevalence reported in non-immigrant children [16]. *B. hominis*, *E. coli* and *G. duodenalis* infections are related to ingestion of food or water contaminated by faeces, and are confirmed as the most frequent parasites among underprivileged people [17]. The number of children with growth rates below normal standard values indicates the persistence of poverty among immigrants and a higher risk of being parasitized by one or more species, particularly by *B. hominis*, although its pathogeneticity is often underestimated [8,17,18].

The close relationship between housing and parasitism confirms that socio-economic conditions significantly compromise health status, and may favour environmental faecal contamination and interpersonal transmission of direct-cycle parasites [18,19] even in a developed country.

In this study, it is difficult to clearly show if parasites were acquired locally or were imported. However, the fact that 9.3% of children were still affected even after a longer stay in Italy - particularly the children living in shacks - suggests that local transmission of parasites cannot be ruled out, especially if poor sanitary conditions persist.

Certainly, improving socio-economic conditions may safeguard children from intestinal parasitism as confirmed by the fact that in this study the time spent in Italy (over a year) appeared as a “protective factor”, because the risk of parasite infections decreased after one year of residence.

Our study shows that immigrant children may be at risk for parasites even in a developed country. In addition, it seems that parasites may interfere with children’s growth, and more in-depth investigations are needed in this direction. Athough no conclusive association may as yet be provided [6], studies must be carried out to investigate biochemical and nutritional markers among children.

Most parasites detected here are listed in the WHO’s *Neglected Disease* and the present study shows that intestinal parasites are not confined to developing countries, highlighting poverty, social exclusion and deprivation in a developed country.

The persistence of parasitism in children and the related risk factors in a developed country indicate an urgent need for extensive improvements to the social and economic conditions of immigrants [20].

## Competing interests

The authors declare that they have no competing interests.

## Authors’ contributions

LM: examined children, collected samples and performed parasitological analysis. FB helped in designing the study, planning the work, interpreting data and drafting the manuscript. DDC: participated in parasitological analysis and data elaboration. LE: participated in the designing the study and examining children’s clinical status. GC: carried out statistical analysis of the data and participated in drafting the manuscript. DO: participated in analysis and interpretation of data and commented on the manuscript. AG: conceived the paper and took part in designing the study, interpreting the data, and in drafting and revising the paper. All authors reviewed and approved the manuscript.
